# Emerging roles for the ADAMTS-like family of matricellular proteins in cardiovascular disease through regulation of the extracellular microenvironment

**DOI:** 10.1007/s11033-024-09255-5

**Published:** 2024-02-07

**Authors:** Karoline Bjarnesdatter Rypdal, Suneel S. Apte, Ida G. Lunde

**Affiliations:** 1 KG Jebsen Center for Cardiac Biomarkers, Institute for Clinical Medicine, University of Oslo, Oslo, Norway; 2 Oslo Center for Clinical Heart Research, Department of Cardiology Ullevaal, Oslo University Hospital, Oslo, Norway; 3 Department of Biomedical Engineering, Cleveland Clinic Lerner Research Institute, Cleveland, OH USA

**Keywords:** Extracellular matrix, Growth factor signaling, Cardiac fibrosis, Microfibrils, Heart failure, Aortic aneurysms

## Abstract

Dysregulation of the extracellular matrix (ECM) occurs widely across cardiovascular pathologies. Recent work has revealed important roles for the «a disintegrin-like and metalloprotease domain with thrombospondin-type 1 motifs like” (ADAMTSL) family of secreted glycoproteins in cardiovascular tissues during development and disease. Key insights in this regard have come from naturally occurring gene mutations in humans and animals that result in severe diseases with cardiovascular manifestations or aortopathies. Expression of ADAMTSL genes is greatly increased in the myocardium during heart failure. Genetically modified mice recapitulate phenotypes of patients with ADAMTSL mutations and demonstrate important functions in the ECM. The novel functions thus disclosed are intriguing because, while these proteins are neither structural, nor proteases like the related ADAMTS proteases, they appear to act as regulatory, i.e., matricellular proteins. Evidence from genetic variants, genetically engineered mouse mutants, and in vitro investigations have revealed regulatory functions of ADAMTSLs related to fibrillin microfibrils and growth factor signaling. Interestingly, the ability to regulate transforming growth factor (TGF)β signaling may be a shared characteristic of some ADAMTSLs. TGFβ signaling is important in cardiovascular development, health and disease and a central driver of ECM remodeling and cardiac fibrosis. New strategies to target dysregulated TGFβ signaling are warranted in aortopathies and cardiac fibrosis. With their emerging roles in cardiovascular tissues, the ADAMTSL proteins may provide causative genes, diagnostic biomarkers and novel treatment targets in cardiovascular disease. Here, we discuss the relevance of ADAMTSLs to cardiovascular medicine.

## Cardiovascular disease is a major cause of morbidity and mortality worldwide

**Fig. 1:**
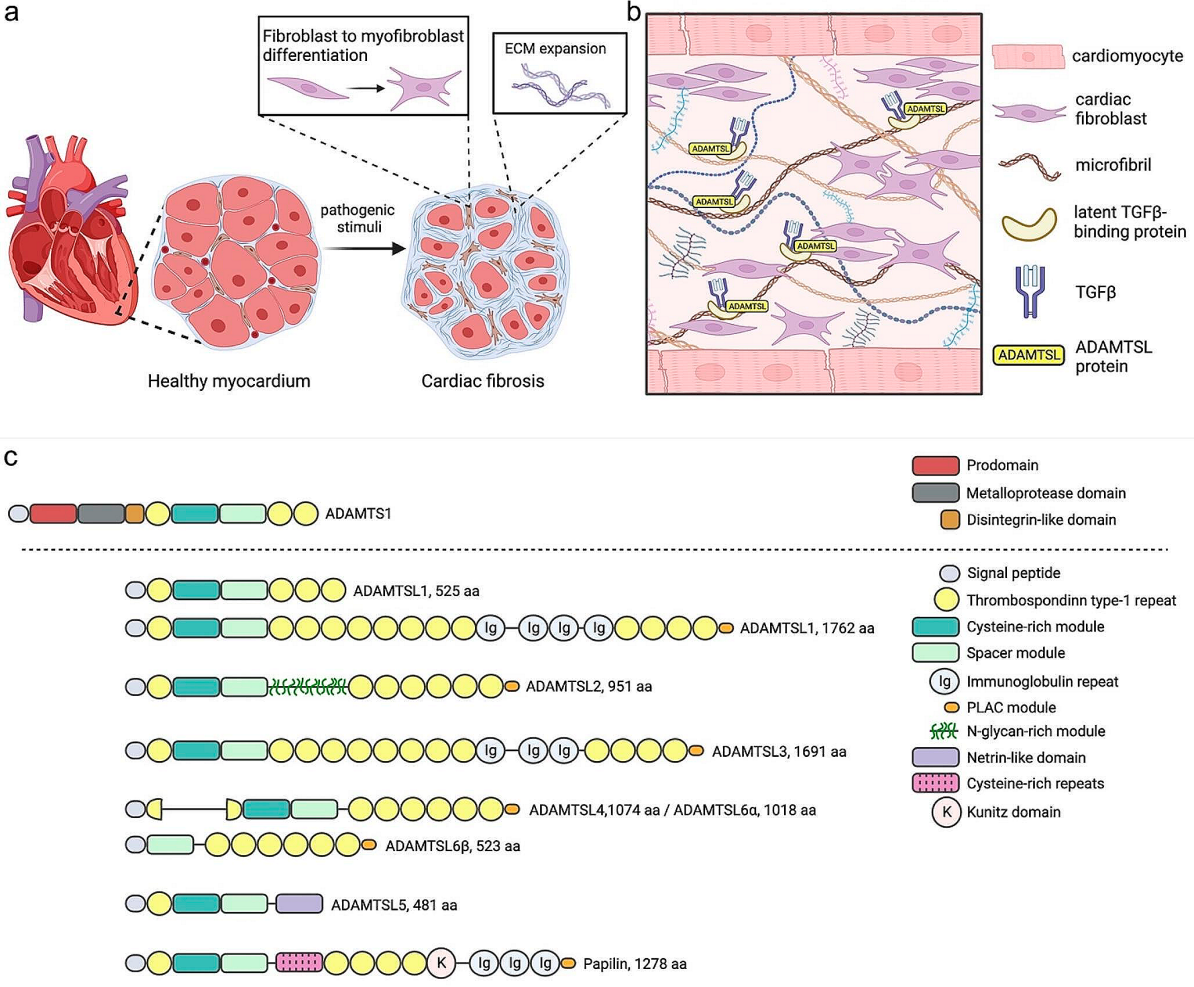
The ADAMTSL family of secreted glycoproteins is part of the cardiac extracellular matrix (ECM). **a**) Pathogenic stimuli to the heart, such as inflammatory cytokines, myocardial stretch, or cardiomyocyte death, trigger cardiac fibroblast to myofibroblast differentiation and ECM expansion, resulting in cardiac fibrosis. **b**) The ADAMTSL protein family members reside in the myocardial ECM, tethered to fibrillin microfibrils and latent TGFβ-binding proteins (LTBPs), and potentially, other ECM macromolecular complexes such as collagen fibrils and basement membranes. **c**) Domain structure of the seven ADAMTSL family members. They share structural similarity with the ancillary domains of the ADAMTS proteases, illustrated here with the structure of ADAMTS1, but lack protease domains (adapted from Apte SS. J Biol Chem. 2009 [12]). Created with BioRender.com

Cardiovascular disease (CVD) is the leading cause of death worldwide, taking over 19 million lives in 2020, and leading to monetary costs higher than for cancer [1]. Abnormal remodeling of the extracellular matrix (ECM), resulting in ECM expansion during a reparative or reactive process, is commonly observed across CVDs, and is termed fibrosis (Fig. 1a). Cardiac fibrosis develops when injury or pathological stimuli such as myocardial stretch, cardiomyocyte death, or neurohumoral and cytokine signaling trigger differentiation of resident cardiac fibroblasts into highly contractile and proliferating myofibroblasts that produce excessive amounts of ECM proteins. Fibrosis stiffens the myocardium and vasculature, preventing their normal function, and ultimately leads to organ failure [2]. Cardiac fibrosis predicts poor patient outcomes and is an independent risk factor for mortality [2]. Management of fibrosis is described as the largest unmet medical need in heart failure today [2] and a deeper understanding of its underlying mechanisms is needed to develop novel therapies. Beyond the heart, optimal vascular rigidity and compliance are dependent on a healthy connective tissue and thus, ECM remodeling is relevant to many disorders, including aortic aneurysms, pulmonary hypertension, small vessel aneurysms and varicose veins.

### ADAMTSL proteins are emerging as molecular players of importance in cardiovascular disease and fibrosis

The cardiovascular ECM is crucial for maintaining tissue integrity. Molecular analyses of extracellular microenvironments have pointed to a seven-membered family of ECM proteins named “a disintegrin-like and metalloprotease domain with thrombospondin-type 1 motifs like” (ADAMTSL) as potential regulators of cardiovascular ECM and fibrosis (Fig. 1b) [3]. Recent evidence strongly reinforces this hypothesis. Recently, a genome-wide association study (GWAS) of UK Biobank participants identified *ADAMTSL1* as one of eleven loci associated with myocardial fibrosis [4]. Moreover, ADAMTSL2 was identified among nine predictive blood biomarkers for adverse outcomes in patients with heart failure [5], and as an independent biomarker for fibrosis in the liver [6]. Studies in humans and mice link ADAMTSL proteins mechanistically to vascular remodeling, heart failure and fibrosis [6–8]. Functional cell culture analyses have repeatedly supported a role for ADAMTSLs in regulation of the pro-fibrotic transforming growth factor (TGF)β [7–11]. The time has come for recognition of ADAMTSL proteins in CVD and fibrosis. Here, the current literature on their role in the cardiovascular system is reviewed, with emphasis on TGFβ signaling and fibrosis in CVD.

### TGFβ is a central regulator of the cardiovascular ECM in development, health, and disease

**Fig. 2:**
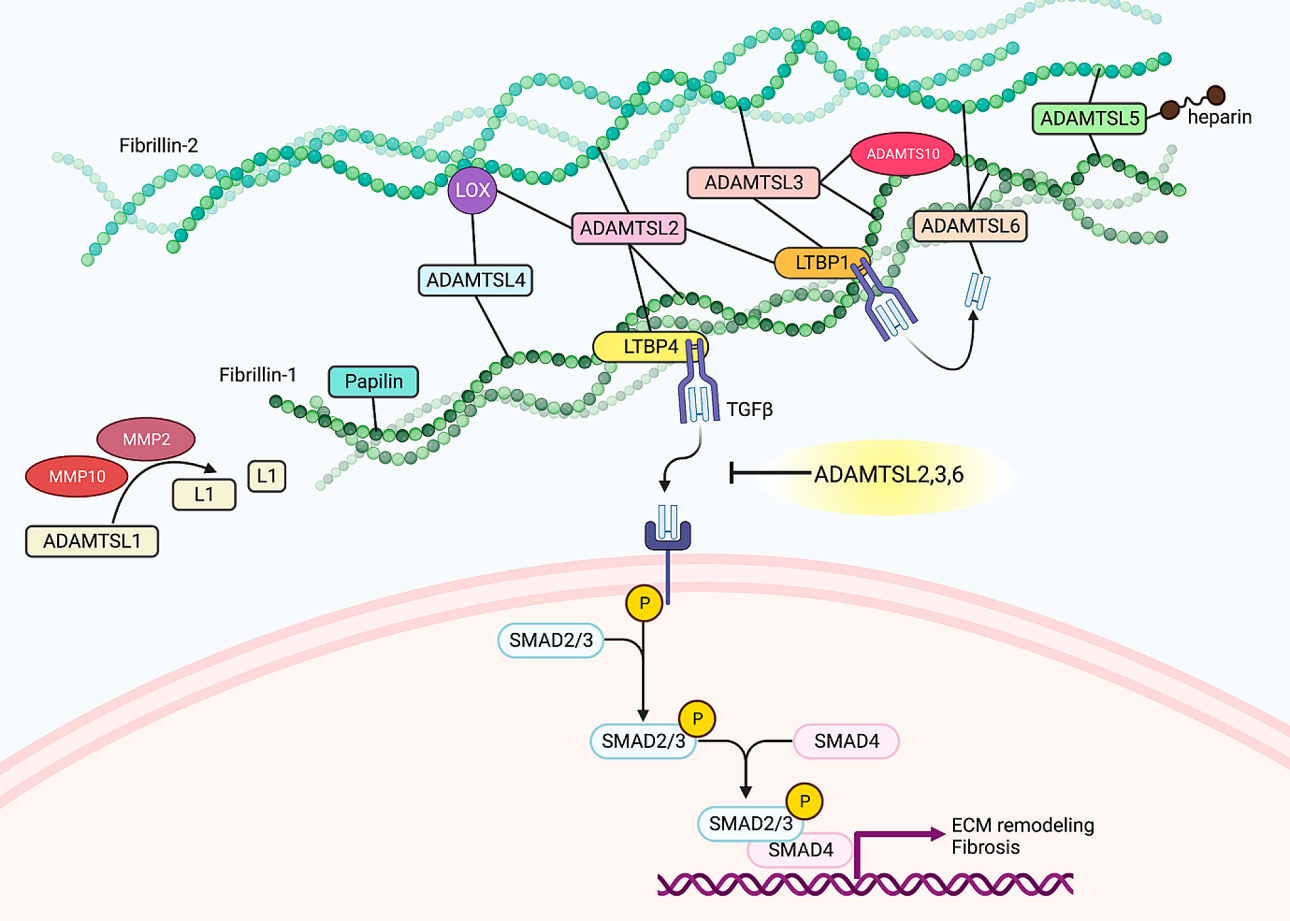
The ADAMTSL interactome. The ADAMTSL proteins reside in the cardiovascular extracellular matrix (ECM) where they bind components of the microfibrils (fibrillin-1 and fibrillin-2) and latent TGFβ-binding proteins (LTBPs). The ADAMTSL proteins have known interactions with the ECM-residing enzymes MMPs, LOX and ADAMTS10, and bind heparin, suggesting an interaction with heparan sulfate proteoglycans. A proposed molecular mechanism of ADAMTSLs in the ECM is shown, whereby ADAMTSLs control the activation of TGFβ, and consequent initiation of the canonical TGFβ-SMAD2/3 signaling cascade. TGFβ signaling leads to transcription of genes promoting ECM remodeling and fibrosis. Created with BioRender.com

The TGFβ superfamily is implicated in major cellular processes from development to disease. Practically every cell of the human body expresses receptors for, or produces, TGFβ, emphasizing its essential roles [13]. TGFβ is stored in the ECM as an inactive complex. First linked to the latency associated peptide (LAP), forming the small latent complex (SLC), and later bound to one of three latent TGFβ binding proteins (LTBPs), together comprising the large latent complex (LLC). The LLC is typically tethered to fibrillin microfibrils and fibronectin fibers in the ECM (Fig. 1b), and interaction between TGFβ and its receptors is inhibited when the LLC is structurally intact. TGFβ can be proteolytically activated by ECM proteases or by physical force on LAP by a_v_ integrins [13]. Active TGFβ initiates canonical signaling through phosphorylation of SMAD2/3 and SMAD4 transcription factors (Fig. 2), or alternatively, non-canonical pathways, leading to diverse cellular responses. In cardiac fibrosis, canonical signaling leads to activation of resident cardiac fibroblasts that differentiate into highly proliferating and ECM-synthesizing myofibroblasts (Fig. 1a). Because of its major role in fibrosis, TGFβ signaling is an appealing treatment target in CVD. Mutations in genes of the canonical signaling pathway, i.e., *TGFB1/2/3*, *TGFBR1/2*, and *SMAD2/3/4*, result in CVDs such aortic aneurysms and aortic dissections, congenital heart defects, cardiac arrythmias, and heart failure [14]. They are typically autosomal dominant and demonstrate the importance of TGFβ signaling in cardiovascular development and disease.

### The ADAMTSL family of matricellular proteins is expressed in cardiovascular tissues

Quantitatively, the cardiac ECM constitutes mainly of collagen I and III fibers. Elastic fibers further provide energy storage for tissue flexibility, recoil and resilience. The fibrillin microfibrils are templates for elastic fiber assembly and docking stations for latent cytokines and growth factors, providing a reservoir of TGFβ that can be readily activated (Fig. 1b). The ECM also includes a range of non-structural, dynamically expressed proteins termed matricellular proteins, and bioactive protein fragments termed matrikines [15]. They typically have high turnover and serve as cofactors for interactions between signaling molecules and surface receptors. Injury or disease triggers expression of matricellular proteins to facilitate tissue remodeling, and they can be activated or degraded by ECM modulating enzymes, e.g., lysyl oxidase (LOX), matrix metalloproteinases (MMPs), and a disintegrin-like and metalloprotease domain with thrombospondin-type 1 motifs (ADAMTSs) [12, 15].

The ADAMTSL family, consisting of ADAMTSL1-6 and papilin, are recognized as important components of the matrisome [12] (Fig. 1c). In mammals, the ADAMTSLs are part of the ADAMTS superfamily, however, the ADAMTSLs lack the key domains associated with ADAMTS protease activity, i.e., the propeptide, catalytic domain and disintegrin-like domains. They appear to have emerged by gene duplication accompanying the evolution of increasing ECM complexity in vertebrae, and are not splice variants of ADAMTS protease genes. The ADAMTS proteases have various ECM regulatory functions including proteolytic processing of collagens, proteoglycans and growth factors. Indeed, they appear to have as significant a role in ECM turnover as MMPs [12]. In contrast, there is limited knowledge of the molecular functions of ADAMTSL proteins. Evidence from the last two decades have strongly suggested a role in regulation of fibrillin microfibrils and growth factor signaling. Genetic variants link ADAMTSLs to connective tissue disorders, cardiac developmental defects, and CVD, with dysregulated TGFβ signaling as a prominent feature [7, 8, 10]. The Genotype-Tissue Expression (GTEx) database from human donors shows that the ADAMTSL family is expressed in human cardiovascular tissues (Table 1). We recently demonstrated ADAMTSL gene upregulation in failing human hearts and showed that overexpression of ADAMTSL2 and ADAMTSL3 inhibited TGFβ signaling in human cardiac fibroblasts [7, 9]. In mice, deletion of ADAMTSL3 resulted in exacerbated cardiac remodeling and failure, with increased TGFβ signaling. These findings indicate important roles for ADAMTSLs in the heart.

### *ADAMTSL* variants are found in connective tissue disorders with cardiovascular manifestations

The ECM is essential for myocardial and vessel homeostasis, and thus, cardiovascular manifestations are frequently observed in connective tissue disorders. Mutations in ECM genes such as *FBN1, COL3A1, ELN, ADAMTS17* and *ADAMTS10* challenge cardiovascular structural integrity [14, 16]. Mutations in *ADAMTSL* genes also challenge cardiovascular physiology. Biallelic *ADAMTSL2* variants results in the connective tissue disorder Geleophysic dysplasia (GD, MIM 231050), which severely affects skeletal growth, muscle, joint and cardiac development, and leads to repeated respiratory infections, heart failure and early death for most patients [10, 14]. The severity is reflected in the *Adamtsl2-/-* mice, which have ventricular septal defects (VSD) and die after birth with severe bronchial occlusion and presumed respiratory failure [11]. Variants in *ADAMTSL6* predispose for aortic aneurysms, which are reproduced in *Adamtsl6+/-* mice [8]. Importantly, a consistent finding in patients with genetically reduced levels of ADAMTSL2 and ADAMTSL6 is a cardiovascular phenotype with increased levels of active TGFβ [8, 10, 14].

### ADAMTSL proteins regulate fibrillin microfibrils and control TGFβ activation

Several studies support a role for ADAMTSL proteins as inhibitors of TGFβ in the ECM [7, 9, 17, 18] (Fig. 2). The molecular mechanism relates to fibrillin microfibrils, where ADAMTSL proteins colocalize with the LLC [19] (Fig. 1b). The ability to bind fibrillin microfibrils was demonstrated for all ADAMTSL family members, except ADAMTSL1. Most ADAMTSLs bind fibrillin-1, and several also bind fibrillin-2, as well as microfibril-associated molecules e.g., LTBPs, TGFβ, ADAMTS10, heparin and LOX [17, 19–23] (Fig. 2). ADAMTSL2, ADAMTSL4 and ADAMTSL6 have further been shown to direct microfibril assembly [11, 17, 22, 24]. Dysregulation of growth factor signaling is a central feature of ECM pathologies and, supporting a functional link, a common consequence of variants in both microfibril and ADAMTSL genes [16]. Increased TGFβ activity is observed in cells and tissues from *Fbn1* and *Adamtsl* knockout mice [7, 8, 11], and ADAMTSL2, ADAMTSL3 and ADAMTSL6 negatively regulate amounts and activity of TGFβ in human cells [7, 9, 17]. The human pathologies associated with ADAMTSL proteins, their connection to CVD, and the currently described animal models are summarized in Table 1. In the following section, we discuss the ADAMTSL members separately in a CVD context.

**Table 1:** Human pathologies, cardiovascular expression levels, protein interactions and animal model phenotypes of the ADAMTSL protein family

Gene	Human genetic disorder	Expression in cardiovascular tissue (median transcripts per kilobase million (TPM), data from GTEx [25])				Protein interactions	Animal model	Animal phenotype
		Heart (LV, *n* = 432)	Heart (AA, *n* = 429)	Aorta (*n* = 432)	Coronary artery (*n* = 240)			
*ADAMTSL1*		0.17	0.64	15.44	3.65	MMP10, MMP2 [26]	Mouse *Adamtsl1*-/- [27]	Knockout mice are healthy and fertile ( [27] and Apte S.S., unpublished) with onset of muscle atrophy from adulthood [27]. No cardiovascular phenotype is described.
*ADAMTSL2*	Geleophysic dysplasia (MIM 231050), Al Gazali skeletal dysplasia (MIM 601356)	3.55	54.51	3.13	13.66	Fibrillin-1/2 [11], LTBP1 [10], LTBP4 [23], LOX [20]	Mouse *Adamtsl2*-/- [11]	Knockout mice die after birth from bronchial occlusion, characterized by peribronchial fibrillin-2 accumulation, and increased TGFβ signaling [11]. Ventricular septal defect was observed.
							Beagle dog founder mutation, Musladin Lueke syndrome [28]	Carrier dogs are unaffected, but dogs homozygous for the R221C mutation have pronounced stiff skin, short stature, typical facies and joint contractures [28]. Collagen-rich fibrotic lesions surround the heart and other organs [18].
*ADAMTSL3*	Tetrasomy 15q [29], 15q25 microdeletion syndrome (MIM 614294)	2.54	9.66	37.80	8.06	Fibrillin-1/2, LTBP1, ADAMTS10 [19]	Mouse *Adamtsl3*-/- [7]	The knockout mice have normal fertility and lifespan, with a skeletal development phenotype of longer tibia [7]. They display abnormal cardiac development with increased heart size [7]. Following cardiac pressure overload, the knockouts show reduced cardiac function, cardiac dilatation and increased TGFβ activity [7]. Juvenile mutant mice have a transient gait anomaly which resolves in adulthood (Martin D.R., Apte S.S., et al. unpublished).
							Aberdeen Angus cattle *ADAMTSL3* founder mutation [30]	Calves homozygous for an *ADAMTSL3* deletion are born with distal joint contractures and joint hyperlaxity which recovers, and have poor muscle development. Adult homozygous mutants have a higher frame score than unaffected littermates [30].
*ADAMTSL4*	Ectopia lentis et pupillae (MIM 225200), Isolated ectopia lentis (MIM 225100)	15.63	21.86	45.58	40.02	Fibrillin-1 [22], LOX [20]	Mouse nonsenseallele *Adamtsl4*^*tvrm267*^ [31]	Homozygous *Adamtsl4*^*tvrm267*^ mice recapitulate ectopia lentis of humans and exhibit focal retinal pigment epithelium (RPE) defects primarily in the inferior eye [31].
*ADAMTSL5*		10.66	9.44	12.92	6.48	Fibrillin-1/2, heparin [21]	Mouse deletion allele *Adamtsl5*^tm1b(EUCOMM)Hmgu^	The International Mouse Phenotyping Consortium (IMPC) reports that knockouts are viable and fertile.
*ADAMTSL6*	Aortic aneurysm, familial thoracic (MIM 619825)	13.11	10.58	30.31	1.94	Fibrillin-1/2 [24], TGFβ1 [17]	Mouse *Adamtsl6*+/- [8]	Heterozygous knockout mice have a normal lifespan with normal fertility [8]. They show progressive thoracic aortic dilation from six months of age and have microfibril alterations with increased TGFβ signaling [8].
*PAPLN*		1.07	4.70	5.04	11.96	Fibrillin-1 [19]	*C. elegans, D. melanogaster* [32]	Abnormal muscle development and reduced survival [32].

### The *ADAMTSL1* locus is linked to cardiac fibrosis in a large human population study, yet its function remains largely unknown

First named Punctin according to its punctate deposition in the ECM, human ADAMTSL1 was characterized 20 years ago [33]. Mature punctin is a short splice variant from the *ADAMTSL1* locus, while a predicted, longer isoform has a 68% sequence identity to *ADAMTSL3*, suggesting shared functions (Fig. 1c). ADAMTSL1 is widely expressed, especially in fibroblasts [25]. Strong skeletal muscle expression was reported in humans and mice [33], and strong aortic expression suggests a role in cardiovascular tissue. Indeed, *ADAMTSL1* variants are enriched in intracranial aneurysms [34], and ADAMTSL1 is downregulated in abdominal aortic aneurysms [34, 35]. ADAMTSL1 is susceptible to proteolysis by MMP10 and MMP2, resulting in ∼20–40 kDa fragments of unknown function [26] (Fig. 2). The *Adamtsl1-/-* mouse has progressive muscle atrophy [27] (Table 1). No cardiovascular phenotype is reported, but TGFβ target genes are differentially expressed [27]. *ADAMTSL1* expression increases in hearts of mice with experimental heart failure and fibrosis [9], linking ADAMTSL1 to cardiac ECM remodeling. Strong evidence for a role in human cardiac fibrosis comes from the recent UK Biobank GWAS where *ADAMTSL1* was discovered among eleven genetic loci linked to cardiac fibrosis [4], including in patients with diabetes mellitus, renal disease, aortic stenosis, cardiomyopathy, heart failure, atrial fibrillation, and rheumatoid arthritis. This study clearly underlines ADAMTSL1 as a biologically relevant molecule in cardiac fibrosis.

### ADAMTSL2 is critical for cardiac development and regulates cardiac TGFβ activity

The ADAMTSL2 protein was characterized in 2007 [36] with a variable molecular mass of 110–150 kDa attributed to glycosylation [36] (Fig. 1c). ADAMTSL2 expression is strongest in the heart (Table 1), with high expression also in lung, nerves, arteries, liver, kidneys and other endocrine organs [25]. In human fetal tissue, ADAMTSL2 is expressed in heart, skin, lung and vasculature [10]. Biallelic *ADAMTSL2* human mutations cause the severe disease GD. GD, and other subtypes of the musculoskeletal disorders collectively known as acromelic dysplasias [16] arise from recessive mutations in several fibrillin-binding proteins i.e., *LTBP3* (GD3, MIM 617809), *ADAMTS10* (Weill-Marchesani syndrome (WMS1), MIM 277600), *ADAMTS17* (WMS4, MIM 613195), *LTBP2* (WMS2, MIM 614819), or dominant mutations in *FBN1* (Acromicric dysplasia, MIM 102370; GD2, MIM 614185; WMS2, MIM 608328) [16]. Half of GD cases are caused by *ADAMTSL2* mutations, and the other half by *FBN1* mutations. Fewer than 1% of GD cases result from *LTBP3* mutations. The overwhelming majority of *FBN1* mutations cause Marfan syndrome [37], but variants in the TGFβ-binding-protein-like domain 5 (TB5) of *FBN1* can result in Acromelic dysplasias [38]. Marfan syndrome has a clinical phenotype of long limbs, arachnodactyly and hypermobile joints [37], while the phenotype of Acromelic dysplasias are quite the opposite, with short stature, brachydactyly, stiff joints and thick skin. Paradoxically, increased TGFβ signaling is observed in both conditions, attributed to dysregulated TGFβ sequestration and/or activation. Cardiovascular defects, events, or heart failure are features of both Marfan syndrome and Acromelic dysplasias [16], demonstrating the importance of adequate TGFβ regulation in cardiovascular tissues. In GD, cardiovascular defects including pulmonary, mitral and aortic valve stenosis, and ventricle and papillary hypertrophy are progressive and manifest through the lifetime. Functional analysis of GD-specific missense *ADAMTSL2* variants shows that the protein is produced, but not secreted, due to misfolding [10]. ADAMTSL2 mutations can also give rise to the more severe Al-Gazali skeletal dysplasia, with cardiovascular defects of pulmonary artery hypoplasia and stenosis, aortic stenosis and perimembranous VSD [39]. The molecular mechanisms of ADAMTSL2 have not been fully elucidated. The largest body of evidence supports a role for ADAMTSL2 in suppression of TGFβ activity, as ADAMTSL2 localizes to microfibrils and binds directly to fibrillin-1 and fibrillin-2 in cultured fibroblasts [11]. ADAMTSL2 also binds LTBP1 [19, 38] and LTBP4 [23], suggesting it is part of a TGFβ sequestering complex (Fig. 2). Increased TGFβ levels and activity are consistently reported in cells from GD patients or experimentally mutated *ADAMTSL2* [10, 23, 38], while overexpression of ADAMTSL2 in cardiac fibroblasts reduces TGFβ activation [9]. However, in the context of increased ADAMTSL2 levels, supplying active TGFβ to cell culture medium increases TGFβ signaling [9]. ADAMTSL2 seems to be part of the sophisticated TGFβ feedback system as TGFβ upregulates ADAMTSL2 expression [9, 40]. ADAMTSL2 interacts with LOX, which crosslinks collagen fibers and is pro-fibrotic [20]. Lower ADAMTSL2 protein levels were observed in *Lox*-/- tissue and tissue from mice treated with the LOX inhibitor β-aminopropionitrile [20]. This could reflect a post-transcriptional regulation of ADAMTSL2 by LOX, which was suggested by similar *Adamtsl2* mRNA levels in WT and *Lox*-/- tissue. On the basis of an observed interaction of LOX and ADAMTSL2 in the secretory pathway, it was speculated that LOX may act as a chaperone required for ADAMTSL2 secretion, or regulate its stability in the ECM [20].

ADAMTSL2 was recently identified as a circulating biomarker of fibrosis in patients with non-alcoholic fatty liver disease (NAFLD), with superior diagnostic performance compared to standard-of-care fibrosis risk-scores [6]. This links ADAMTSL2 to liver fibrosis, with possible implications for other fibrotic diseases, and is supported by its upregulation in cardiac fibrosis [9, 40]. Interestingly, ADAMTSL2 was recently identified as a predictor of prognosis and adverse outcomes in patients with heart failure [5], which could be related to degree of cardiac fibrosis. In support of this, a founder mutation in *Adamtsl2* causes Musladin-Lueke syndrome in beagles, characterized by severe skin and intermuscular fibrosis (Table 1). The disorder resembles human stiff skin syndrome (MIM 184900), which is caused by *FBN1* mutations [28]. The Musladin-Lueke dogs develop a dense collagen network surrounding the organs, and isolated fibroblasts have a myofibroblast phenotype with high levels of TGFβ [18], thus linking ADAMTSL2 to TGFβ regulation and fibrosis in multiple species across several organ systems, including the heart.

### ADAMTSL3 is cardio-protective in the failing heart

ADAMTSL3 was recognized as an ADAMTSL1/punctin-1 paralog and thus named punctin-2 at the time of discovery [41]. N-linked glycosylation contributes significantly to its observed mass of 210 kDa [41] (Fig. 1c). ADAMTSL3 shows high expression in the heart and vasculature (Table 1), in addition to skeletal muscle, lung, kidney, neurons, skin and liver [25, 41]. Like ADAMTSL2, ADAMTSL3 binds the N-terminus of fibrillin-1 and the C-terminus of LTBP1 [19]. With unknown consequence, ADAMTSL3 also binds the C-terminus of ADAMTS10 [19]. ADAMTS10 cleaves fibrillin-1 and fibrillin-2, but with low efficacy, leading to a net increase in microfibril assembly [42]. *ADAMTS10* and *FBN1* mutations both cause WMS, characterized by thick skin, joint contractures and perturbed TGFβ regulation. The WMS *FBN1* variants disrupt the binding sites for ADAMTSL2, ADAMTSL3, ADAMTSL6 and papilin on fibrillin-1 [19], suggesting they all contribute to the disease. ADAMTSL3 may form a larger complex with the LLC and ADAMTS10 on microfibrils (Fig. 2), potentially affecting ADAMTS10 activity. Multiple studies have demonstrated an important role for ADAMTSL3 in growth, with consistent correlation of human *ADAMTSL3* variants with height and lean body mass [43, 44]. The *ADAMTSL3 Bos Taurus* sequence shares great similarity with human *ADAMTSL3*, and interestingly, mutated *ADAMTSL3* in cattle leads to bovine arachnodactyly, also known as “fawn calf syndrome”, a recessive connective tissue disorder that resembles human congenital contractual arachnodactyly, which is caused by *FBN2* mutations (MIM 121050) [30] (Table 1). The phenotypic consequences of *ADAMTSL3* variants suggest a growth-limiting function. *Adamtsl3*-/- mice reflect the growth and cardiac phenotype, with increased heart weights and longer tibiae [7] (Table 1).

*ADAMTSL3* variants are enriched in patients with intracranial aneurysms, and ADAMTSL3 expression is reduced in both intracranial and abdominal aortic aneurysms [34, 35]. Extra copies of *ADAMTSL3* have been observed in some patients with a rare duplication of the distal arm of chromosome 15, known as Tetrasomy 15q25 [29]. *ADAMTSL3* over-dosage is suggested as the key mediator of heart defects in these patients, as patients presented with complex cardiac malformations, related to high mortality, only when ADAMTSL3 was part of the duplication [29] (Table 1). Cardiac defects were also reported in patients with a heterozygous chromosomal microdeletion on 15q25 that includes *ADAMTSL3* [45]. ADAMTSL3 levels increase in human and mouse heart failure, suggesting a role in cardiac function [7]. Indeed, *Adamtsl3*-/- mice subjected to experimental heart failure by pressure overload develop exacerbated contractile dysfunction and cardiac dilatation with increased mortality (Table 1). Reduced cardiac function in *Adamtsl3*-/- mice was attributed to ECM dysregulation with increased TGFβ activity, myofibroblast differentiation and collagen crosslinking, which together impaired myocardial function. ADAMTSL3 overexpression in cultured human cardiac fibroblasts results in reduced TGFβ activity and myofibroblast differentiation, as well as reduced collagen deposition [7], together indicating a cardioprotective role for ADAMTSL3.

### ADAMTSL4 is upregulated in failing hearts and its function is largely unknown

The *ADAMTSL4* gene was characterized in 2003 [46], then named thrombospondin type 1 repeat containing gene (TSRC1). ADAMTSL4 shares a structural homology with ADAMTSL6α (Fig. 1c), and due to post-translational modifications the ADAMTSL4 protein varies from 150 to 250 kDa in size [22]. ADAMTSL4 is widely expressed in the mammalian eye [22], with high expression also in nerves, female reproductive organs, bladder, lung and vasculature [25]. ADAMTSL4 colocalizes with fibrillin-1 in the ECM, enhances fibrillin-1 deposition [22], and interacts with LOX [20], suggesting a role in ECM crosslinking (Fig. 2). *ADAMTSL4* variants are strongly associated with eye pathology. Biallelic *ADAMTSL4* mutations cause ectopia lentis et pupillae (MIM 225200) and isolated ectopia lentis (MIM 225100) [47] (Table 1). Ectopia lentis is also caused by variants in *FBN1, ADAMTS10, ADAMTS17* and *LTBP2*, suggesting a functional relationship [48] in the regulation of zonule assembly. *ADAMTSL4* variants phenocopy TGFβ receptor mutations in craniosynostosis [49], supporting a role in TGFβ regulation. During experimental heart failure in mice, cardiac ADAMTSL4 levels increase [9], indicating a role in the failing heart. Interestingly, in a recent observational study, *ADAMTSL4* variants were associated with protection from coronary artery disease in two cohorts of high-risk patients [50], and pathogenic variants in *ADAMTSL4* were reported in spontaneous coronary artery dissection [51]. Thus, although little is known, there are clear indications that ADAMTSL4 has a role in vascular disease.

### ADAMTSL5 is highly expressed in cardiovascular tissues with potentially unique functions in the pericellular ECM

ADAMTSL5 was characterized in 2013, with a unique C-terminal netrin-like domain, resulting in the shortest ADAMTSL protein of 55–60 kDa [21] (Fig. 1c). The linker upstream of the netrin-like module is targeted by proteases, released, and binds to heparin in the ECM [21]. Human *ADAMTSL5* shows high expression in female reproductive organs, nerves, aorta and the heart [25] (Table 1), and mouse *Adamtsl5* is widely expressed, including in skin, skeletal muscle, brain, lung, kidney, and heart tissue [21]. Mechanistically, ADAMTSL5 binds fibrillin-1 and fibrillin-2 and localizes to microfibrils in cell culture (Fig. 2), however, it was not shown to affect fibrillogenesis in vitro [21]. Being described only ten years ago, ADAMTSL5 has not been extensively studied. The presence of the netrin-like domain suggests unique functions. Similar to the other family members, ADAMTSL5 is upregulated in experimental heart failure [9], and the high expression in cardiovascular tissues suggests an important role in the heart and vasculature.

### ADAMTSL6 determines vascular integrity via regulation of TGFβ

In 2010, two isoforms of ADAMTSL6, ADAMTSL6α and ADAMTSL6β, were identified (Fig. 1c) [24]. Both isoforms are transcribed from the *ADAMTSL6* gene (also designated *THSD4*) in humans. Human *ADAMTSL6* is highly expressed in esophagus, female reproductive organs, arteries, and the heart [25] (Table 1). In mice, ADAMTSL6α expression was observed in all tissues examined, while ADAMTSL6β was selectively expressed during embryogenesis, and in adult brain, spinal cord, eye, kidney, stomach, and uterus. Of note, the strongest expression for ADAMTSL6α was seen in the adult heart [24]. Protein analysis of ADAMTSL6α and ADAMTSL6β showed 145 kDa and 95 kDa proteins, respectively, detected in the ECM of arteries, skin, tendons, cartilage, and mitral valves of the heart [24]. Mechanistically, ADAMTSL6β binds to the N-terminus of fibrillin-1, and both ADAMTSL6α and ADAMTSL6β promote fibrillin-1 fibrillogenesis [24]. ADAMTSL6β was also shown to bind and inhibit active TGFβ [17] (Fig. 2). In recent years, *ADAMTSL6* variants were linked to aortic dilatation and aneurysms [8, 52, 53]. Most convincingly, 22 likely pathogenic heterozygous variants were identified across *ADAMTSL6* in a cohort of 1114 unrelated patients with thoracic aortic aneurysms [8]. Molecular analysis of six variants revealed ECM disorganization with increased TGFβ activity in aortic tissue, as well as aberrant fibrillin-1 deposition in vitro [8]. *Adamtsl6*+/- mice have normal lifespans, with increasing aortic diameter, ECM disorganization and progressive loss of smooth muscle cells from six months age [8] (Table 1). These findings are supported by two independent GWAS, showing association of *ADAMTSL6* variants with aorta size [52], blood pressure, spontaneous coronary artery dissection, and aortic dilation [53]. Reduced *ADAMTSL6* expression correlates with increased TGFβ activity and fibroblast activation in tumor tissue [54], in line with studies linking ADAMTSL6 to regulation of TGFβ, emphasizing the important role of ADAMTSL6 in TGFβ regulation.

### Papilin inhibits collagen assembly and facilitates collagen degradation

Papilin was isolated from *Drosophila* cell culture medium in 1987 as a large 900 kDa glycoprotein localized to basement membranes [55]. Papilin-deficient *Drosophila* larvae show abnormal muscle development and reduced survival, and papilin is essential for survival in *C. elegans* [32]. Mechanistically, *Drosophila* papilin noncompetitively inhibits the procollagen N-proteinase ADAMTS2 in vitro [32], while mammalian papilin binds fibrillin-1, suggesting a similar role to other ADAMTSL family members [19] (Fig. 2). An ECM regulatory role for papilin has been demonstrated, where it facilitates collagen IV removal during growth in *C. elegans* and is abundant in the growing basement membrane [56]. Interestingly, knock-down of *C. elegans Papilin* restricts growth and results in fibrotic accumulation of collagen IV [56], suggesting anti-fibrotic properties of papilin (Tabel 1). Human papilin is less studied but is reportedly smaller than its *Drosophila* homolog (Fig. 1c). In humans, *PAPLN* is highly expressed in nerves, female reproductive organs, thyroid and spleen, but modestly expressed in the cardiovascular system [25] (Table 1). A study of patients with NAFLD supports a possible role for papilin in human fibrosis, as its expression increased with advancement of disease and fibrosis progression [57], similar to ADAMTSL2 [6]. Although expression is low in human cardiovascular tissues, expression may increase upon stress, as was observed in experimental heart failure in mice [9]. The potential collagen-regulating role of papilin needs further investigation in human cardiovasculature.

### ADAMTSL proteins are potential therapeutic targets in cardiovascular disease

**Fig. 3:**
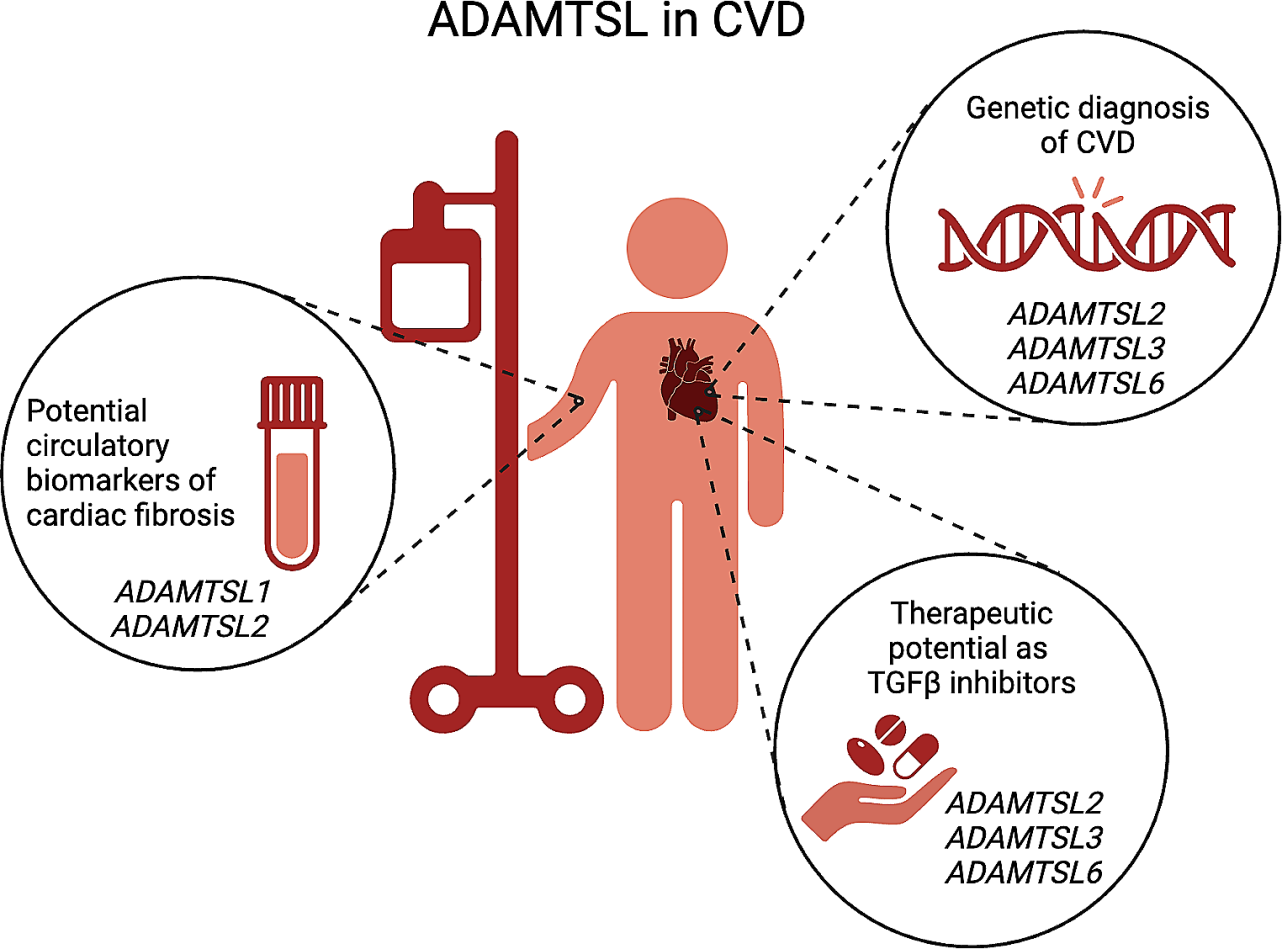
Future directions for ADAMTSL proteins as potential genetic substrates, biomarkers, and therapeutic targets in cardiovascular disease. *ADAMTSL*2, *ADAMTSL3* and *ADAMTSL6* genetic variants have been identified in diseases of the heart and vasculature. Biallelic *ADAMTSL2* variants cause the rare and deadly syndromes Geleophysic dysplasia and Al-Gazali dysplasia with several cardiac manifestations [10]. *ADAMTSL3* is involved in rare chromosomal disorders with cardiovascular malformations [29, 45]. *ADAMTSL6* heterozygous mutations give rise to aortic aneurysms [8]. ADAMTSLs have potential as circulatory biomarkers of cardiac fibrosis. ADAMTSL2 is a demonstrated biomarker of liver fibrosis [6] and predicts adverse events in heart failure [5], and ADAMTSL1 is independently associated with cardiac fibrosis in heart failure [4]. ADAMTSL proteins may be therapeutic targets to limit TGFβ activity in the heart and vasculature by overexpression or recombinant peptide delivery. Created with BioRender.com

The currently best understood therapeutic potential of ADAMTSL proteins in CVD is related to their role in reduction of TGFβ activity. Targeting TGFβ is a focus area in development of anti-fibrotic treatment. Preclinical studies show beneficial effects of directly inhibiting TGFβ in different pathologies, with e.g., monoclonal antibodies, but also report adverse outcomes [58], likely due to the critical physiological roles of TGFβ. Thus, new strategies to target TGFβ pathways are warranted. While there is a long way towards therapeutic application of ADAMTSL proteins, their TGFβ inhibitory effects extend to several diseases of the cardiovascular and connective tissues. In vitro findings of anti-fibrotic effects by ADAMTSL overexpression or recombinant proteins [7, 9, 17] hold promise for in vivo benefits, however this avenue of research has yet to be explored, involving administration of ADAMTSL proteins or peptides, or genetic overexpression, in model organisms (Fig. 3).

After increased TGFβ signaling was shown to drive CVD progression in Marfan syndrome, studies in mice revealed that TGFβ inhibition could ameliorate the cardiovascular phenotype [37]. *ADAMTSL6* variants phenocopy the aortic manifestations of Marfan syndrome, with increased TGFβ signaling and progressive aortic dilatation and dissection [8], indicating a shared molecular pathway. The therapeutic role of ADAMTSL6 was accordingly tested in a Marfan mouse model with truncated fibrillin-1. Most intriguingly, administration of ADAMTSL6β rescued the disorganized microfibrils and activation of TGFβ [17], demonstrating a therapeutic effect. This was further validated in isolated cells from Marfan patients, restoring the microfibril network and reducing TGFβ signaling in a dose-dependent manner [17]. The therapeutic effect of ADAMTSL6 in aneurysms may be extended to other CVDs with overactive TGFβ, such as aortopathies and fibrosis, and warrants further investigation. There is also evidence of therapeutic TGFβ inhibition by ADAMTSL2 in cardiovascular tissue. Lack of ADAMTSL2 in the ECM is the underlying cause for GD, with a cardiac phenotype and molecular pathology related to increased TGFβ activity [10], as well as the cause for tissue fibrosis in beagle dogs [18], while significantly associated with human fibrosis of the liver [6] ADAMTSL2 may be an important player in development of cardiac fibrosis, as levels increase considerably in the myocardium and circulation of patients and mice during heart failure development [5, 9, 40]. Encouraging a potential therapeutic role, increased levels of ADAMTSL2 reduce fibrotic signaling in cultured cardiac fibroblasts [9]. Anti-fibrotic effects are also demonstrated for ADAMTSL3, which reduces TGFβ signaling, myofibroblast differentiation and type I collagen synthesis in cardiac fibroblasts [7]. In line, ADAMTSL3 is cardio-protective and improves survival in mouse heart failure [7]. Although not as extensively studied as ADAMTSL2 and ADAMTSL6, ADAMTSL3 seems to harbor similar molecular properties in the cardiovascular microfibril niche and may be a therapeutic target in CVD (Fig. 3).

Mechanistically, the ADAMTSL proteins likely regulate TGFβ both directly and indirectly. ADAMTSL2 and ADAMTSL3 bind to the LLC [19], and are therefore suggested to stabilize inactive TGFβ in the ECM. ADAMTSL6 binds active TGFβ directly [17], suggesting a direct inhibition in the ECM. All ADAMTSL proteins, except for ADAMTSL1, have been shown to bind fibrillin microfibrils [19], and may affect TGFβ signaling indirectly by regulating the microfibril network in which the LLC is sequestered. However, the precise molecular mechanisms remain to be elucidated. A functional relationship between ADAMTSLs and their sister proteases, the ADAMTSs, was anticipated, as the ancillary domain of ADAMTS is crucial for substrate recognition. However, this has only been demonstrated for papilin [32]. Further evidence of an ADAMTSL regulatory role vis-á-vis ADAMTS proteases is lacking, yet some ADAMTS enzymes have demonstrated roles in CVD related to regulation of TGFβ. ADAMTS4 can bind and cleave the LLC, and its inhibition reduces cardiac fibrosis in mice through reduced TGFβ signaling [59]. Similar functions was observed for ADAMTS16, which increases cardiac fibrosis through TGFβ-induced fibroblast activation [60]. It remains to show whether ADAMTS and ADAMTSL proteins might compete for LLC binding sites. Mutations in *ADAMTS10* and *ADAMTS17* phenocopy mutations in *ADAMTSL4* (ectopia lentis) [48] and *ADAMTSL2* (acromelic dysplasias) [16], suggesting common functional pathways, and ADAMTSL3 also binds directly to ADAMTS10 [19]. These proteins may form functional complexes that work in synergy with microfibrils to balance levels of active growth factors in the cardiac ECM. Although regulation of TGFβ may be a shared function in the ADAMTSL family, genetic evidence demonstrate unique functions of different ADAMTSL proteins (Table 1). This may be related to their individual domain structures, where ADAMTSL1 (long form) share sequence homology with ADAMTSL3, and ADAMTSL4 is homologous to ADAMTSL6α, while ADAMTSL2 and ADAMTSL5 contains unique protein sequences (Fig. 1c). Their function may also be related to their specific spatiotemporal expression. While they are all present in the heart and/or vasculature the degree of expression varies (Table 1), which may be related to cell type, developmental stage, or pathophysiological conditions [9, 25].

### Future directions of ADAMTSL studies in cardiovascular disease

Circulating biomarkers reflecting cardiac fibrosis are urgently needed to aid in diagnosis, prognosis, and therapeutic guidance. As differential levels of ADAMTSL2 in blood samples predict the onset and degree of liver fibrosis [6], efforts should be made to investigate ADAMTSL2 as blood biomarker of cardiac fibrosis, and possibly also other ADAMTSL molecules (Fig. 3). Indeed, a recent machine-learning omics-based analysis of circulating proteins associated with poor outcomes in patients with heart failure identified ADAMTSL2 as one out of nine predictive proteins. Repeat measurements in a patient cohort showed that circulating ADAMTSL2 levels were already raised two years prior to the study’s adverse patient outcomes [5]. Such studies provide motivation to investigate the functions of ADAMTSL molecules in cardiovascular health and disease, which still have a long way to go.

The current body of evidence strongly suggests ADAMTSLs as growth factor regulators, and their role seems to extend beyond TGFβ inhibition. Future studies should aim to identify their interactome and reveal other ADAMTSL effects. Translational studies are important and in vivo experiments will be essential to unravel their role in CVD. No preclinical models have yet explored potential therapeutic effects of ADAMTSLs in CVD by increasing their levels. As large, highly modified proteins, full-length recombinant ADAMTSLs are challenging to produce. ADAMTSL fragments may be more readily made, and feasibility of production by viral vectors has been shown. In experimental heart failure, ADAMTSL2 and ADAMTSL3 are interesting candidates for *in vivo* overexpression, either as a transgenic mouse model, or via viral injections. Generation of active ADAMTSL small peptides may facilitate development of clinically relevant molecules.

A fraction of the genetic substrate for CVD has hitherto been found, and identification of individual patterns of genetic loci that cause CVD, so-called polygenic risk scores, will aid in precision or personalized medicine. The emergence of large-scale genetic analysis for diagnostic work-up, will likely reveal more causative gene networks that may include *ADAMTSL*s. *ADAMTSL* mutations have already been found to cause rare, recessive disorders with cardiovascular involvement, and progression was recently made with the identification of *ADAMTSL6* as a causative gene in aortopathies [8]. Future publications will likely support this finding with further discoveries of *ADAMTSL6* variants in patients, and potentially, variants in other *ADAMTSL* genes (Fig. 3).

## Concluding remarks

Accumulating evidence support important roles for ADAMTSL proteins in regulation of the cardiovascular microfibril niche with consequences for ECM assembly, growth factor activation and signaling. ADAMTSL2 appears to have specific functions oriented toward regulation of the fibrillin-LTBP-TGFβ complex in the ECM. Insights from analysis of ADAMTSL2 in GD patients, heart failure patients, mouse models and cell cultures may suggest general principles applicable to ADAMTSLs whose function remains as yet undefined. However, additional and possibly unrelated roles are evident from human *ADAMTSL* gene variants and the distinctive phenotypes arising from experimental gene inactivation. Differing phenotypes between mouse ADAMTSL models likely result from evolution of distinct protein domain structures, spatio-temporal regulation and intermolecular interactions. As multimodular proteins, ADAMTSLs could function in diverse molecular networks. Several ADAMTSLs are emerging as important in CVD, i.e., *ADAMTSL1* locus in cardiac fibrosis, *ADAMTSL6* genetic variants in aorthopathies, ADAMTSL2 in GD and heart failure, ADAMTSL4 in coronary artery dissection and ADAMTSL3 in mouse heart failure, where they represent opportunities for therapeutic intervention. In particular, their ability to limit TGFβ activity is medically relevant. Another aspect is their potential as circulating biomarkers, where ADAMTSL2 may be specifically promising for cardiac fibrosis and heart failure. Elucidation of their individual molecular mechanisms and translational potential for diagnosis, prognosis and therapy will be critical to determine their value in future CVD medicine.

## Data Availability

No datasets were generated or analysed during the current study.

## Abbreviations

ADAMTSL: a disintegrin-like and metalloprotease domain with thrombospondin-type 1 motifs like
CVD: Cardiovascular disease
GD: Geleophysic dysplasia
GWAS: Genome-wide association study
ECM: Extracellular matrix
LLC: Large latent complex
LTBP: Latent TGFβ-binding protein
NAFLD: Non-alcoholic fatty liver disease
TGFβ: Transforming growth factorβ
VSD: Ventricular septal defect
WMS: Weill-Marchesani syndrome
